# A Rare Case of Diffuse Alveolar Hemorrhage Caused by Fentanyl-Laced Marijuana

**DOI:** 10.7759/cureus.38523

**Published:** 2023-05-04

**Authors:** Quang Le, Gulshan Dangol, Amit Bhandari

**Affiliations:** 1 Department of Internal Medicine, Hospital Sisters Health System (HSHS) St. John's Hospital, Springfield, USA; 2 Department of Internal Medicine, Good Samaritan Regional Health Hospital, Mount Vernon, USA

**Keywords:** pulmonary hemorrhage, dah, diffuse alveolar hemorrhage, marijuana-laced fentanyl, fentanyl, illicitly manufactured fentanyl

## Abstract

Synthetic fentanyl adulteration has become a significant threat to public safety. It is commonly mixed into other drugs of abuse to lower costs and increase its addictive potential. Diffuse alveolar hemorrhage (DAH) is a rare but life-threatening complication associated with the use of fentanyl-laced products. Given the current trend, we anticipate an increase in the incidence of DAH. It is crucial to recognize and treat DAH early in its course for better outcomes. We present a case of DAH due to an overdose of marijuana laced with fentanyl, manifesting as hemoptysis, and provide a review of the current literature on the topic.

## Introduction

Synthetic opioids, including fentanyl, have emerged as a major public health challenge over the past decade [1]. Although heroin-involved deaths decreased by 7% from 2019 to 2020 in the US, synthetic opioid-involved deaths increased by 56%. Fentanyl belongs to the phenylpiperidine class and can be 80 to 100 times more potent than morphine [1,2]. Due to its highly potent analgesic effect, it is frequently diverted for abuse. Its overdose carries many dangerous complications, including centrally mediated respiratory depression, non-cardiogenic pulmonary edema, and very rarely diffuse alveolar hemorrhage (DAH), all leading to cardiopulmonary arrest. Importantly, it is not detected by routine immunoassay urine drug testing, which might pose a significant diagnostic challenge for many clinicians [3]. It is important to educate clinicians about the dangerous association between fentanyl-laced products and DAH so that we can recognize and treat DAH early for better outcomes.

## Case presentation

**Figure 1:**
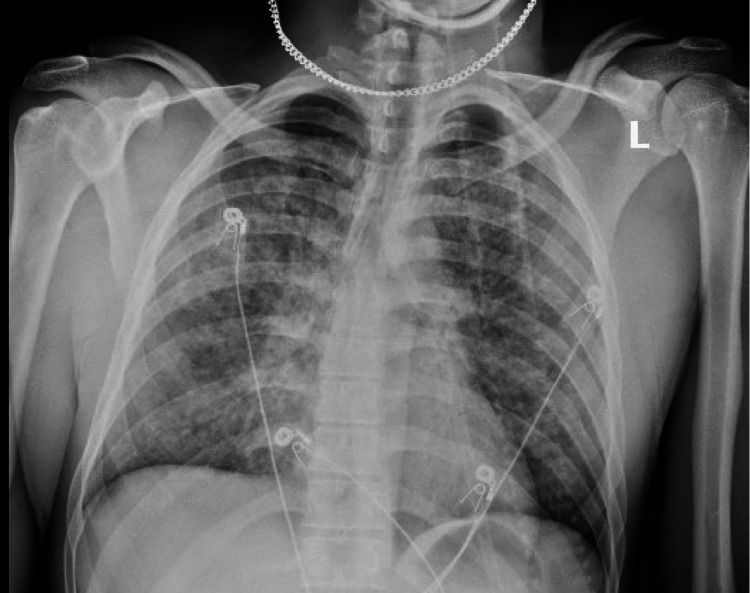
Chest X-ray on day one with diffuse bilateral infiltrates.

A 31-year-old man with a past medical history of depression and chronic low back pain was brought to the emergency room after being found unresponsive at his friend's house. Physical examination at the scene was significant for pinpoint pupils and a Glasgow Coma Scale score of 4 points. Two milligrams of intravenous naloxone drastically improved his mentation. Later, he admitted to smoking only marijuana. He acknowledged occasional marijuana use but adamantly denied any history of cocaine or opioid abuse. While in the emergency room, he developed tachypnea, worsening hypoxia with pulse oximetry (SpO_2_) of 85%, and coughed up dark red blood, approximately 200 milliliters. Chest X-ray demonstrated diffuse bilateral infiltrates (see Figure 1).

Arterial blood gas analysis on a non-rebreather mask revealed pH 7.0, carbon dioxide (CO_2_) 46 mmHg, oxygen (O_2_) 90 mmHg, bicarbonate (HCO_3_) 20 mEq/L, and arterial O_2_ partial pressure to fractional inspired O_2_ ratio (PaO_2_/FiO_2_) of 90. He was emergently intubated. The urine toxicology screen was positive only for marijuana and negative for other substances including cocaine. Complete blood count, comprehensive metabolic panel, and urinalysis were all unremarkable. Autoimmune workups were negative for anti-nuclear antibodies, anti-neutrophil cytoplasmic antibody titer, rheumatoid factor, and anti-Jo1 antibodies (Table 1).

**Table 1:** A synopsis of major work-ups and results MCV: Mean corpuscular volume; MCHC: Mean corpuscular hemoglobin concentration; RDW: Red cell distribution width; ANA: Antinuclear antibody; ANCA IFA: Antineutrophil cytoplasmic antibodies; Ab: Antibody; IgG: Immunoglobulin G; BAL: Bronchoalveolar lavage; BUN: Blood urea nitrogen; ALP: Alkaline phosphatase; ALT: Alanine aminotransferase; AST: Aspartate aminotransferase

Complete Blood Count		Metabolic Panel	
WBC	11.4 k/uL	Sodium	136 mmol/L
RBC	4.44 g/dL	Potassium	3.4 mmol/L
Hemoglobin	13.8 g/dL	Chloride	91 mmol/L
Hematocrit	40.9	CO2	21 mmol/L
MCV	92.1	BUN	10 mg/dL
MCHC	31.1	Creatinine	1.4 mg/dL
RDW	12.8	Glucose	227 mg/dL
Platelet count	148	Albumin	4.8 g/dL
Neutrophils %	90	ALP	135 U/L
Autoimmune Labs		ALT	26 U/L
ANA	Negative	AST	42 U/L
ANCA IFA	<1:20	Bilirubin	0.5 mg/dL
Anti JO-1 Ab IgG	Negative		
Rheumatoid Factor	<10		
Anti GBM Ab IgG	Negative		
BAL Fluid Analysis			
Color	Red		
RBC	105.500 /uL		
Nuclear Cells	2225 /uL		
Lymphocyte	1%		
Monocyte	12%		
Neutrophil	85%		
Eosinophil	0%		

**Figure 2:**
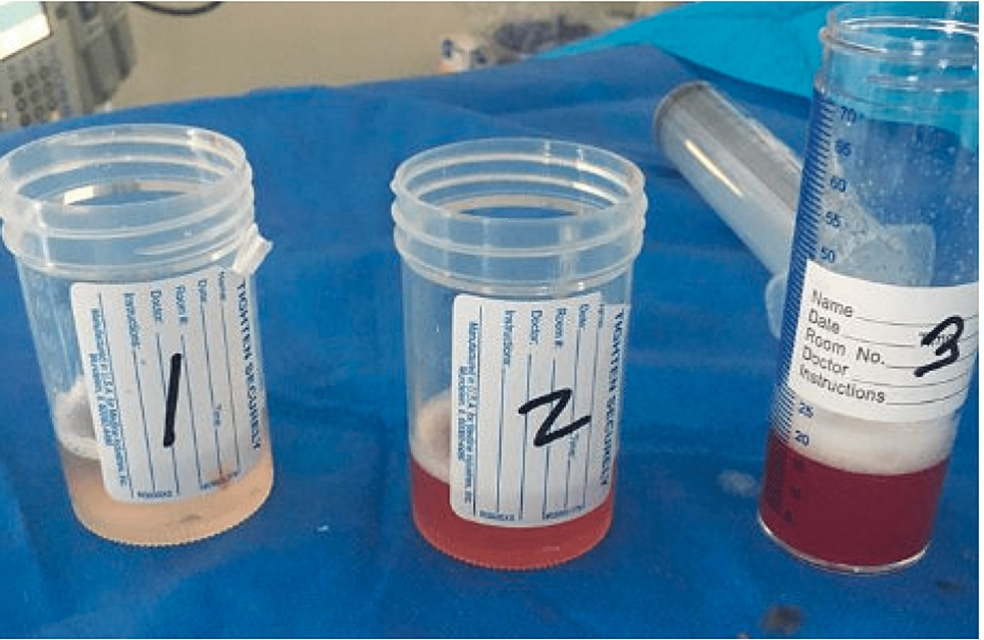
Sequential BAL with progressively bloodier lavages. BAL: Bronchoalveolar lavage

The Hepatitis panel and HIV tests were negative. Bronchoscopy with bronchoalveolar lavage (BAL) showed 106,500 red blood cells/uL with sequential fluid lavages being progressively bloodier, which confirmed the diagnosis of DAH (Figure 2).

**Figure 3:**
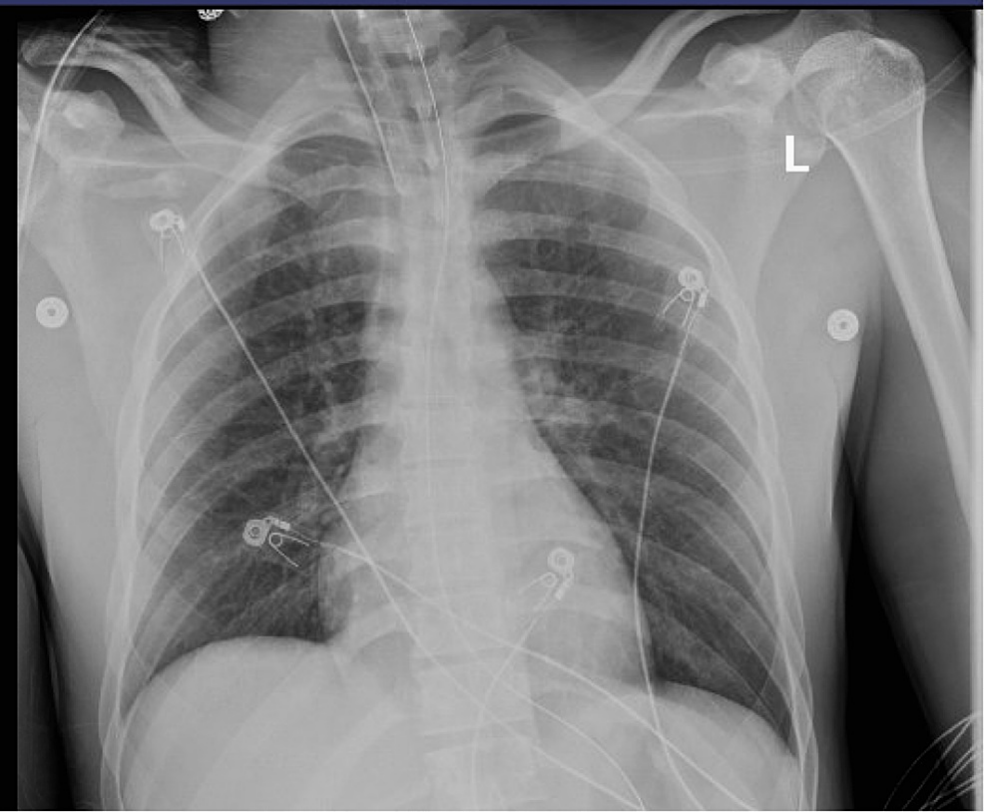
Chest X-ray showed near complete resolution of infiltrates on day three.

Bacterial culture, fungus culture, and pneumocystis stain on BAL, as well as the acid-fast culture of BAL specimen, were negative. BAL cytology showed macrophages, neutrophils, and blood. Echocardiogram revealed an ejection fraction (EF) of 65% with normal diastolic and valvular functions. A fentanyl metabolite panel using the first serum sample drawn upon presentation to the ER confirmed fentanyl levels of 2.3 ng/mL and nor-fentanyl of 1.45 ng/mL. He was managed mainly with supportive care on ventilation. A repeat chest X-ray on day three showed dramatic improvement (Figure 3).

He was extubated on day four. He was later discharged in stable condition and continued to do well at a follow-up visit a week later.

## Discussion

Illicitly manufactured fentanyl (or clandestinely produced fentanyl) has become a rising threat to public health in the United States. Notably, it is often adulterated with other substances, leading to various forms of abuse, ranging from intravenous to oral, nasal, and smoking routes. Diffuse alveolar hemorrhage is a life-threatening complication of fentanyl-laced product inhalation. It is caused by bleeding from alveolar capillaries and manifests as hemoptysis, acute anemia, diffuse lung infiltration, and acute respiratory failure. It is diagnosed early by bronchoscopy with bronchoalveolar lavage [4]. Etiologies of DAH include connective tissue disease, small vessel vasculitis, infection, and often cocaine abuse [4]. Pulmonary hemorrhage is well-known in crack cocaine abuse, with an approximate incidence of 5.7% [5]. However, upon literature review, there are only a few cases of DAH related to opioid overdose [6-8]. In our case, a secondary workup ruled out other potential causes of DAH, including autoimmune diseases and infections. BAL revealed a large amount of blood with progressively bloodier on sequential lavages. The initial urine drug screen in our patient was negative for cocaine and opioids. However, testing of the first serum sample using the "Liquid Chromatography/Tandem Mass Spectrometry Method" showed elevated fentanyl levels than the normal range of 0.63-2.0 mg/mL. We decided to send out the test due to initial clinical suspicion though it is not a routine practice in the ER setting.

The precise mechanism of opioid-induced alveolar hemorrhage is unknown. Some consider it an extreme form of acute lung injury leading to non-cardiogenic pulmonary edema due to severe endothelial disruption causing capillary bleeding [6]. Two primary mechanisms leading to acute lung injury in opioid abuse may involve the antagonist naloxone and negative-pressure barotrauma. The first theory suggests that administering naloxone too quickly can cause rapid precipitation of opioid withdrawal, leading to a massive increase in blood catecholamines. This phenomenon, like amphetamine or cocaine usage, significantly increases cardiac afterload, directly injures pulmonary endothelial cells, and severely constricts pulmonary circulation [7,9]. The second theory involves negative-pressure barotrauma. In this scenario, opioid intoxication causes the glottis to relax backward or even close. When naloxone is administered, its rapid reversal action causes the patient to take a deep breath against the relaxed glottis, generating enormous negative intrathoracic pressure. This creates a pressure gradient drawing fluid into the alveolar space and leading to acute lung injury [7]. There have been case reports of diffuse alveolar hemorrhage caused by diverse types of opioid overdoses, including fentanyl, butyrfentanyl, and heroin (Table 2). Management includes cessation of implicated drugs, supportive care with respiratory support of oxygenation, and mechanical ventilation if needed.

**Table 2:** Previous case reports of DAH in association with opioid abuse. DAH: Diffuse alveolar hemorrhage

Cases	Patient Info	UDS	Opioids Types	Hb drop	BAL results	Autoimmune work-ups
1	22 y/o, male with hemoptysis [8]	Negative for cocaine	Heroin	14.1- 12.4	Consistent with DAH	Negative
2	34 y/o, male with hemoptysis [8]	Negative for cocaine	Heroin	16.5-12.2	n/a	Negative
3	18 y/o male with hemoptysis and respiratory failure [7]	Negative for cocaine	Butyr-fentanyl	15.5-12.3	Thin red-tinged secretions with subepithelial petechiae and progressive reddening of ﬂuid returns from bronchoalveolar lavage	
4	45 y/o male with loss of consciousness [6]	Negative for cocaine	fentanyl		Frank blood was seen throughout the airway, and gross blood was returned on BAL from the right upper lobe.	Negative

## Conclusions

Diffuse alveolar hemorrhage is a dangerous and potentially life-threatening complication. Fentanyl-laced product abuse can be one of the potential causes. We expect more data and case reports will arise in the near future. Clinicians should be aware of this complication, and a systematic approach to diagnosis and management should be reviewed and implemented accordingly.
